# MSDeepAMR: antimicrobial resistance prediction based on deep neural networks and transfer learning

**DOI:** 10.3389/fmicb.2024.1361795

**Published:** 2024-04-17

**Authors:** Xaviera A. López-Cortés, José M. Manríquez-Troncoso, Ruber Hernández-García, Daniel Peralta

**Affiliations:** ^1^Department of Computer Sciences and Industries, Universidad Católica del Maule, Talca, Chile; ^2^Centro de Innovación en Ingeniería Aplicada (CIIA), Universidad Católica del Maule, Talca, Chile; ^3^Laboratory of Technological Research in Pattern Recognition (LITRP), Universidad Católica del Maule, Talca, Chile; ^4^IDLab, Department of Information Technology, Ghent University-imec, Ghent, Belgium

**Keywords:** MALDI-TOF, deep learning, antibiotic resistance, *Escherichia coli*, *Klebsiella pneumoniae*, *Staphylococcus aureus*, transfer learning

## Abstract

**Introduction:**

Antimicrobial resistance (AMR) is a global health problem that requires early and effective treatments to prevent the indiscriminate use of antimicrobial drugs and the outcome of infections. Mass Spectrometry (MS), and more particularly MALDI-TOF, have been widely adopted by routine clinical microbiology laboratories to identify bacterial species and detect AMR. The analysis of AMR with deep learning is still recent, and most models depend on filters and preprocessing techniques manually applied on spectra.

**Methods:**

This study propose a deep neural network, MSDeepAMR, to learn from raw mass spectra to predict AMR. MSDeepAMR model was implemented for Escherichia coli, Klebsiella pneumoniae, and Staphylococcus aureus under different antibiotic resistance profiles. Additionally, a transfer learning test was performed to study the benefits of adapting the previously trained models to external data.

**Results:**

MSDeepAMR models showed a good classification performance to detect antibiotic resistance. The AUROC of the model was above 0.83 in most cases studied, improving the results of previous investigations by over 10%. The adapted models improved the AUROC by up to 20% when compared to a model trained only with external data.

**Discussion:**

This study demonstrate the potential of the MSDeepAMR model to predict antibiotic resistance and their use on external MS data. This allow the extrapolation of the MSDeepAMR model to de used in different laboratories that need to study AMR and do not have the capacity for an extensive sample collection.

## 1 Introduction

Antimicrobial resistance (AMR) has become one of the most urgent global public health problems (O'Neill, 2016), whose current growth leads to an estimate of an annual death toll of more than ten million annually by 2050, and a cost of approximately 100 trillion USD worldwide (Brogan and Mossialos, 2016; O'Neill, 2016). In general, AMR is the process by which bacteria can survive exposure to antibiotics that, under normal conditions, would be deadly or stop their growth. According to a Nature report (“The Antibiotic Alarm”), antibiotics have been consistently and heavily over-prescribed by doctors worldwide for decades (Nature, 2013). Besides, the indiscriminate use of antibiotics in livestock (Li et al., 2018; Hickman et al., 2021), and the environmental factors that favor the distribution of resistant genes (Lin et al., 2021) have directly contributed to the development of antibiotic resistance.

Antibiotic-resistant mechanisms can be either intrinsic or acquired. In the former, structural or functional characteristics of the bacteria allow them to resist a particular antibiotic. In the latter, bacteria develop resistance to an antibiotic through different mechanisms: (i) minimization of the intracellular concentrations of an antibiotic as a result of poor penetration into the bacterium or as a result of antibiotic efflux; (ii) modification of the antibiotic target by genetic mutation or post-translational modification of the target; and (iii) inactivation of the antibiotic by hydrolysis or modification (Blair et al., 2014).

Regarding AMR detection, the antibiotic sensitivity test (AST) is key in clinical treatments. Testing for antibiotic resistance/susceptibility is typically based on measuring the bacterial growth in the presence of that antibiotic, which takes up to 72 h to obtain results. Hence, new, rapid, and effective techniques are needed to address these challenges.

### 1.1 Mass spectrometry

Mass spectrometry (MS) is a technique that measures the mass/charge ratio *(m/z)* of the atoms or molecules of a sample, after ionizing them. The potential of MS lies in its ability to measure the exact mass of these molecules and to obtain information from the ion fragments of the analyte. MALDI-TOF MS (Matrix-Assisted Laser Desorption/Ionization Time-Of-Flight Mass Spectrometer) is one of the most used techniques in this field (Tanaka et al., 1988). It corresponds to a Desorption Ionization System with Laser Assistance by a Matrix, coupled with the ion analyzer TOF (Time of Flight). MS has had a significant impact in clinical microbiology, allowing for quick identification of bacteria from an intact cell or a whole cell Peptide Mass Fingerprint (PMF) (Singhal et al., 2015). It provides higher accuracy, rapidity, and cost-effectiveness than conventional methods used in microbiology, yielding results in minutes rather than hours (Singhal et al., 2015). This technique has also shown better resolution and reproducibility than gel-based protein or DNA fingerprint techniques (Fenselau and Demirev, 2001; Lay, 2001). The discovery of suitable matrices and the use of whole/intact cells for recording the PMF of bacteria in the mass range of 2–20 kDa, followed by databases for bacterial identification, has made MALDI-TOF MS an excellent alternative for this area. Specifically, “MALDI Biotyper,” developed by Bruker Daltonics, has been considered as a platform to operate and analyze samples with a simple extraction/preparation method (Seng et al., 2009). Since MALDI received regulatory approval from the Food & Drug Administration (FDA) of the United States in 2013, it has been available worldwide for routine identification of cultured bacteria from human specimens (*in vitro* diagnosis). MALDI-TOF MS has rapidly become a reference method for identifying a wide range of microorganisms. Its application for detecting microorganisms such as bacteria has also been widely established, reducing turnaround time and simplifying workflows in clinical microbiology laboratories (Patel, 2015; Welker et al., 2019; Oviaño and Rodríguez-Sánchez, 2021).

These advantages highlight MALDI-TOF MS as a fast, reliable method to identify AMR (Florio et al., 2020), which allows a rapid antibiogram in < 3 h. The methodology for bacterial resistance detection consists of incubating microorganisms with the antibiotic, then centrifugation is performed, and the supernatant obtained is analyzed using MALDI-TOF.

A bacteria is considered to be resistant when an enzyme that degrades the antibiotic [such as carbapenemases and extended-spectrum beta-lactamases (March-Rosselló, 2017)] is detected in its spectrum. On the one hand, the peak corresponding to the mass/charge of the antibiotic disappears. On the other hand, new peaks appear in the spectrum, corresponding to metabolites related to the rupture of the antibiotic. Only the antibiotic peak can be seen in the case of a susceptible (i.e., non-resistant) bacteria. The sensitivity of this experimental technique is close to 100%, which means this method can be used on grown colonies, isolation plates (Lasserre et al., 2015), and grown blood culture bottles from patients (Oviaño et al., 2014). Several methods have been proposed to analyze MALDI-TOF spectra for subspecies discrimination. Some methods focus on visual examination of the spectra to discover strain-specific peaks (Wolters et al., 2011; Lasch et al., 2014), while others are based on the use of ClinProTools software to identify strain-representative peaks (Mather et al., 2016; Villarreal-Salazar et al., 2022).

### 1.2 Machine learning on mass spectrometry

The similarity between MALDI-TOF spectra of highly related strains hinders their visual interpretation (Camoez et al., 2016). Therefore, this analysis involves searching particular, possibly complex patterns in large volumes of data. In this context, the potential of artificial intelligence is very promising (Mather et al., 2016), particularly machine learning techniques.

Machine learning (ML) allows computers to learn without being explicitly programmed for the task at hand. The type of problem and data this research addresses (MALDI spectra with known information about antibiotic resistance) calls for supervised learning algorithms, which are trained using a dataset formed by instances (in this case, each spectrum is an instance), each labeled with a discrete class or a real value (in this case, the resistance/susceptibility of the bacteria). Then, a trained classifier can predict the class of new instances. In recent years, the field of medicine has focused on applying ML-based methods to analyze MS data due to their potential to analyze complex data and the ability to identify biomarkers (Olate-Olave et al., 2021; Tapia-Castillo et al., 2021; López-Cortés et al., 2022; González et al., 2023). Specifically, MS coupled with ML techniques has been widely used in different areas, including health: (i) detection/diagnosis of diseases in humans (Drew et al., 2017), animals (López-Cortés et al., 2017, 2019), among others; (ii) detection of pathogens such as bacteria (Bruyne et al., 2011; Didelot et al., 2012; Dematheis et al., 2022), fungi (Becker et al., 2014; Bolt et al., 2016); and most recently in (iii) AMR prediction (Florio et al., 2020; Huang et al., 2020; Weis C. et al., 2020; Weis et al., 2022; Feucherolles et al., 2022; Wang et al., 2022; Zhang et al., 2022; Guerrero-López et al., 2023).

Recent studies have focused on refining species identification (Guajardo et al., 2022) and determination of AMR (Wang et al., 2018, 2019; Huang et al., 2020; Weis et al., 2022). A recent systematic review (Weis C. V. et al., 2020) has concluded that, despite the number of studies and their quality, there are still some limitations related to poor reproducibility, a small sample size, and a lack of external validation. In this sense, it is necessary to persist in improving the algorithmic techniques used when classifying antibiotic resistance, and this is reflected in the current state of the art, where researchers account for novel and complex classification techniques such as ensemble models (Zhang et al., 2022) or convolutional neural networks (CNN) (Wang et al., 2022). In this reference, a CNN architecture is presented for the identification of *Enterococcus faecium* resistance to Vancomycin, marking a promising research avenue, since CNNs have already been shown to outperform classical ML algorithms on data problems with high dimensionality (LeCun et al., 2015; Lippeveld et al., 2020).

Regarding the study of AMR by using ML approaches, there are different studies with a focus on the use of other experimental techniques such as (i) MS (Wang et al., 2019; Delavy et al., 2020; Huang et al., 2020); (ii) Genome sequencing (Bhattacharyya et al., 2019; Kim et al., 2020); (iii) Infrared microscopy (Sharaha et al., 2019); and (iv) PCR (Athamanolap et al., 2017). Specifically, several works have been focusing on the use of MS coupled to ML in the study of Candida albicans fluconazole resistance detection (Delavy et al., 2020), discrimination of contagious strains of *Streptococcus* (Esener et al., 2018), detection of carbapenem-resistant *Klebsiella pneumoniae* (Huang et al., 2020), and rapid classification of group B of *Streptococcus serotypes* (Wang et al., 2019), among others.

These advances and the increasing prevalence of AMR worldwide highlight the need for efficient techniques to detect bacterial resistance to antibiotics and facilitate the pathogen-directed clinical treatment of the infection. Thus, combining MALDI-TOF with artificial intelligence is an excellent opportunity for this task. It could improve the patient's quality of life and recovery since they would receive timely and direct treatment, also reducing public health costs.

In terms of data availability, a recent study has generated a public database called DRIAMS (Weis et al., 2022), with more than 750,000 antibiotic resistance mass spectra profiles collected in four different laboratories in Switzerland. The study implemented three classification algorithms: logistic regression, LightGBM (Light Gradient Boosting Machine), and a deep neural network (multilayer perceptron). LightGBM presented the best classification results for *E. coli* and *S. aureus*, while the multilayer perceptron obtained the best score for *K. pneumoniae*. These extensive public databases open the way for new and advanced methodologies for AMR analysis to be investigated, as in the present work using deep learning (DL). This methodology is distinguished by its ability to detect new patterns in complex data sets, but it requires a large amount of data to train the models.

In this context, transfer learning (Weiss et al., 2016) has become a hot research topic in many fields, allowing us to start the training from models already pre-trained on large (often publicly available) datasets. These pre-trained models can be fine-tuned with small datasets by laboratories with limited sample collection and computing capacity, which can, in such a way, take advantage of powerful models. A recent proposal in this direction is to detect AMR using deep learning using transfer learning based on whole genome sequence data (Ren et al., 2022). However, to the best of our knowledge, no transfer learning proposals have been made for AMR based on MS techniques.

Our research proposes a complete and novel methodology based on deep learning (DL) and transfer learning for directly analyzing raw MS data to identify antibiotic resistance in three different bacterial species. The use of raw MS data implies a significant reduction of the typical preprocessing (smoothing, baseline correction, peak picking, among others) made with MS data. The dataset for our study was constructed based on DRIAMS (Weis et al., 2022). The bacteria with the highest number of samples and clinical relevance were included: *Escherichia coli, Klebsiella pneumoniae*, and *Staphylococcus aureus*. The set of antibiotics studied for the identification of resistance is detailed in Table 1. First, the data set was formed from the raw mass spectra. Next, the MSDeepAMR model was trained and tested to obtain an area under the receiver-operating characteristic (AUROC). In total, 13 models of antibiotic resistance were implemented with results of AUROC >0.80 in most of the cases studied, showing a 10% improvement over the state-of-the-art. Then, transfer learning was applied to evaluate our models in external databases to study whether laboratories with a lower sample collection capacity can use these models. Our results demonstrate that performing transfer learning substantially improves the evaluation of the model on external data. The MSDeepAMR model generally showed excellent results for classifying antibiotic resistance in different bacterial species.

**Table 1:** Number of samples of each bacterium and antibiotic under study in DRIAMS-A.

**Bacteria**	**Antibiotic**	**Number of samples**	
		**Susceptible**	**Resistant**
*E. coli*	Ciprofloxacin	3.445	1.466
		Ceftriaxone	3.875	1.086
		Cefepime	4.051	839
		Piperacillin-tazobactam	4.449	350
		Tobramycin	4.240	636
*K. pneumoniae*	Ciprofloxacin	2.325	513
		Ceftriaxone	2.411	449
		Cefepime	2.477	362
		Meropenem	2.794	61
		Tobramycin	2.527	319
*S. aureus*	Ciprofloxacin	3.141	616
		Fusidic acid	3.513	253
		Oxacillin	3.064	726

Finally, as was mentioned in previous paragraphs, conventional methods for antibiotic sensitivity tests (AST) take up to 72 h to obtain results. In this way, our approach (MSDeepAMR) can significantly reduce the time in the part of the AST, implying to the health industry a decrease in public costs and an improvement in patients' quality of life. Besides, this research opens the door to integrating MSDeepAMR within the MALDI-TOF device to enable on-the-fly AMR detection due to the network classifying the raw data directly without manual preprocessing.

Considering the proposed methodology and the obtained results, the article's contributions are as follows:

- A systematic and reproducible methodology for antibiotic resistance detection is proposed based on deep neural networks and transfer learning, achieving state-of-the-art results.
- The MSDeepAMR model architecture has been evaluated in several scenarios, demonstrating its ability to predict antibiotic resistance in *E. coli, K. pneumoniae*, and *S. aureus* against different types of antibiotics.
- The proposed methodology performs transfer learning to evaluate reproducibility on external datasets, a pioneering study within the context of MS, highlighting the model improvement and its successful adaptation to external data.

The rest of the paper is structured as follows. Section 2 details the proposed MSDeepAMR methodology, describing experiment settings, transfer learning evaluation, and ablation study. Section 3 presents the results of the conducted experiments. Section 4 discusses the obtained results. Finally, concluding remarks on the study and future works are stated in Section 5.

## 2 Materials and methods

**Figure 1:**
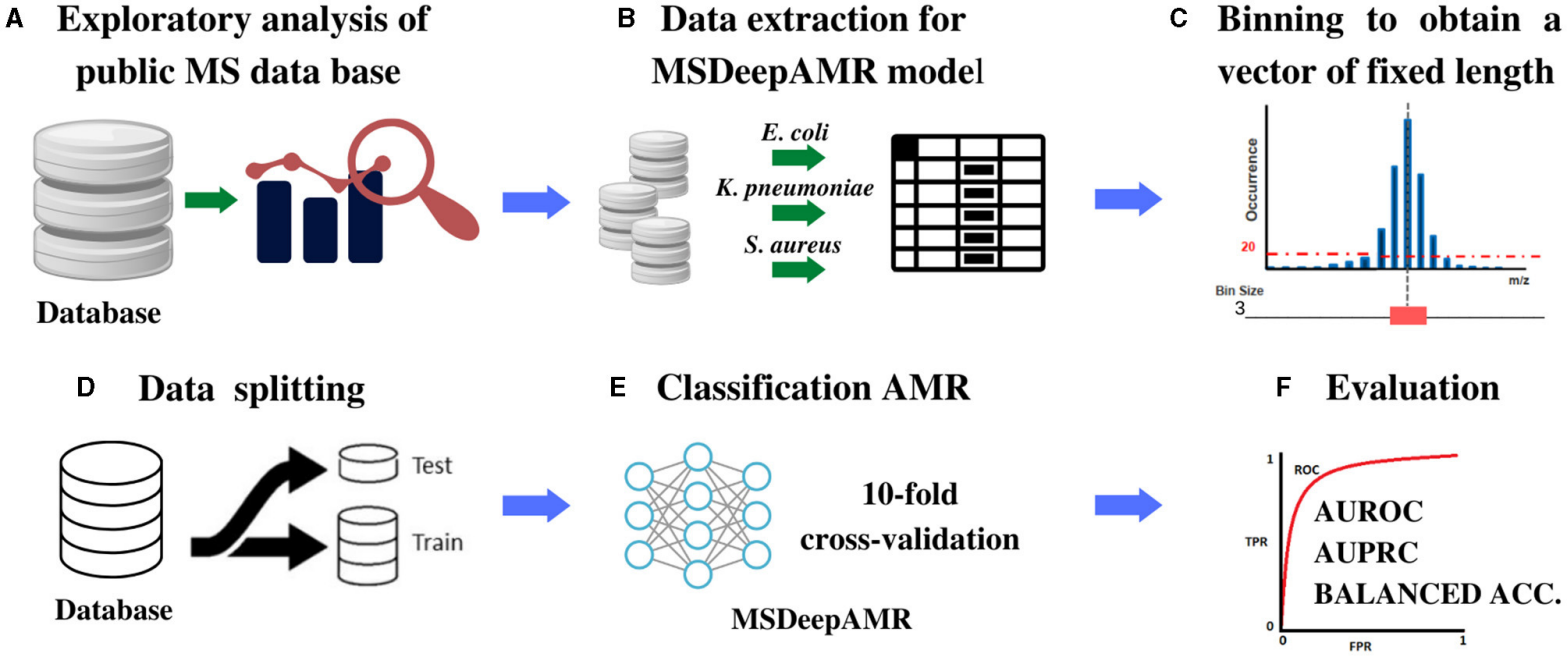
Scheme of the methodology proposed for the identification of AMR. **(A)** MS database selection followed by an exploratory analysis of the content, **(B)** Extraction of the bacteria chosen to be studied, **(C)** Binning of the spectra into equal-sized feature vectors to obtain a DB Adhoc for deep learning models implementation, **(D)** Data split into 80% training and 20% test, stratified by both antimicrobial class and sample case number. **(E)** DL models implementation: For training, ten-fold cross-validation and hyperparameters' optimization was used, and for testing, ten-fold cross-validation was used to evaluate the final model. **(F)** Model performance evaluation and comparison were made according to AUROC, AUPRC, and Balanced Accuracy.

This study implements a DL architecture to identify antibiotic resistance in different bacterial species from raw MS data. As detailed in Figure 1, the first step corresponds to the dataset construction from DRIAMS (Weis et al., 2022), chosen due to its high number of samples. The second step is the extraction of the bacterial data to be used in the present study. In the third step, binned mass spectra are computed to obtain vectors of the same length. Finally, data splitting is performed to train and test the proposed architecture.

### 2.1 Datasets

In the present study, we used the public database DRIAMS (Weis et al., 2022), which has about 300,000 mass spectra of different types of bacteria with more than 750,000 antibiotic resistance profiles. This database consists of four sub-collections (DRIAMS-A, DRIAMS-B, DRIAMS-C, and DRIAMS-D) corresponding to the different clinical laboratories where the samples were collected. DRIAMS-A has the largest number of samples and, therefore, was used to implement and train the MSDeepAMR model, while the remaining ones were used for external testing and transfer learning. Initially, the dataset included 803 different types of bacterial and fungal pathogens. However, given the high number of samples required to train deep neural networks, the following bacteria were selected due to their relevance according to the World Health Organization (WHO) (Asokan et al., 2019) and to their number of samples: *Escherichia coli* (*n* = 5, 000), *Klebsiella pneumoniae* (*n* = 2, 800), and *Staphylococcus aureus* (*n* = 3, 800). These bacteria are on the list of priority pathogens presented by WHO. Table 1 details the number of samples for each class of bacteria and antibiotics under study. Our neural network was trained with raw mass spectra data. A bin size of 3 Da in the range of 2,000 to 20,000 Da was applied. This binning produces a fixed-length vector suitable for the DL algorithms.

### 2.2 Deep learning

This study proposes a deep-learning approach for identifying *E. coli, K. pneumoniae*, and *S. aureus* bacterial species with resistance to different types of antibiotics (Table 1). Thus, the input data corresponds to the raw MS data represented by a total binned vector of 6,000 features. In contrast, the output corresponds to identifying the resistance (class 1 label) or susceptibility (class 0 label) of the given sample to the studied antibiotic.

#### 2.2.1 Model implementation: MSDeepAMR

The MSDeepAMR model was applied to 13 different study cases (Table 1), which includes three of the most clinically relevant bacteria and the most commonly used antibiotics to treat them: *E. coli* (ciprofloxacin, ceftriaxone, cefepime, piperacillin-T., tobramycin), *K. pneumoniae* (ciprofloxacin, ceftriaxone, cefepime, meropenem, tobramycin), and *S. aureus* (ciprofloxacin, fusidic acid, oxacillin). In order to find a structure that would perform well in all the study cases, we took as a starting point the architecture presented in Wang et al. (2022). The architecture of the model was optimized based on the bacteria-antibiotic pair with the highest number of samples (*E. coli*-ceftriaxone), for which a hyperparameter grid search was performed. Subsequently, this architecture was applied to the rest of the bacteria-antibiotic pairs, and each one was optimized in the same way until the final architecture was reached. In this way, the final architecture contains the following parameters:

- The number of convolutional layers (1 to 5).
- The number of filters and kernels for each convolutional layer (filters: 32–256 with a step of 32, kernels: 3–19 with a step of 1).
- The number of fully connected layers (1 to 5).
- The number of neurons within each fully connected layer (32–256 with a step of 32).

**Figure 2:**
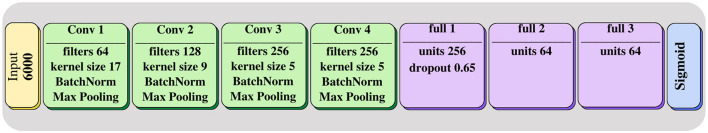
MSDeepAMR architecture: four convolutional layers followed by three fully connected layers. The last layer corresponds to the Sigmoid classifier, which indicates the probability of belonging to one of the classes.

As shown in Figure 2, our model comprises four one-dimensional convolution layers, allowing the network to learn to differentiate the locations of the *m/z* peaks. Additionally, each convolutional block contains a batch normalization layer to reduce the overfitting and a max-pooling layer to reduce dimensionality and focus the attention of the CNN on the *m/z* peaks in each convolution. The classification module consists of four fully connected layers preceded by a dropout layer. The last layer has one output neuron with a sigmoid activation, where the output of each neuron corresponds to the probability that the studied sample presents a resistant or susceptible profile to the antibiotics under study. As for the parameters of the network, the four convolutional layers contain 64, 128, 256, and 256 filters, respectively. The kernel sizes were 17, 9, 5, and 5, and the three fully connected layers before the output layer were composed of 256, 64, and 64 units, respectively. Mean and max pooling were tested, after which mean pooling was selected due to the higher AUROC and AUPRC obtained. The dropout probability was set to 0.65. For training, a maximum of 100 epochs was set in conjunction with early stopping with patience = 4. At the same time, the learning rate of the Adam optimizer was initialized at 10^−4^, with a learning rate reduction of 0.1 when the loss function remained unchanged.

### 2.3 Ablation study

An ablation study was performed to evaluate the behavior of MSDeepAMR when different modifications were applied to the final model. For this purpose, a comparison has been made through a 10-fold cross-validation for each of the study cases (Detailed in Section 2.2.1). Thus, we compared how normalization and regularization layers improve the model performance after the hyperparameter search grid. The evaluation considered three different model modifications:

- Baseline model, without any normalization or regularization layer.
- MSDeepAMR model with batch normalization after each convolutional layer.
- MSDeepAMR final model, with batch normalization after each convolutional layer and dropout after the first fully-connected layer.

### 2.4 External test and transfer learning

Transfer learning (Pan and Yang, 2010) consists of adapting a model trained on a “source” dataset to perform well when applied to a “target” dataset, typically by using a few instances from the target set to fine-tune the pre-trained model. It enables external laboratories with little sample collection capacity to adapt complex models—pre-trained on much larger datasets—to their specific needs (Ebbehoj et al., 2022). The implementation of transfer learning on MALDI-TOF data is a problem that needs to be studied because there are only two mass spectrometry systems that dominate the market: MALDI Biotyper System from Bruker Daltonics and ViteK MS from Biomeriux (Dierig et al., 2015; Hou et al., 2019). Therefore, differences between data collected by two different laboratories with similar sample collection equipment are expected to be limited. Thus, it would facilitate the application of transfer learning techniques to reduce the need to train large models from scratch.

As mentioned above, the DRIAMS database contains three sub-collections of external data with smaller numbers of samples (DRIAMS-B, DRIAMS-C, and DRIAMS-D) corresponding to data collected by different laboratories using the same mass spectrometry system.

**Figure 3:**
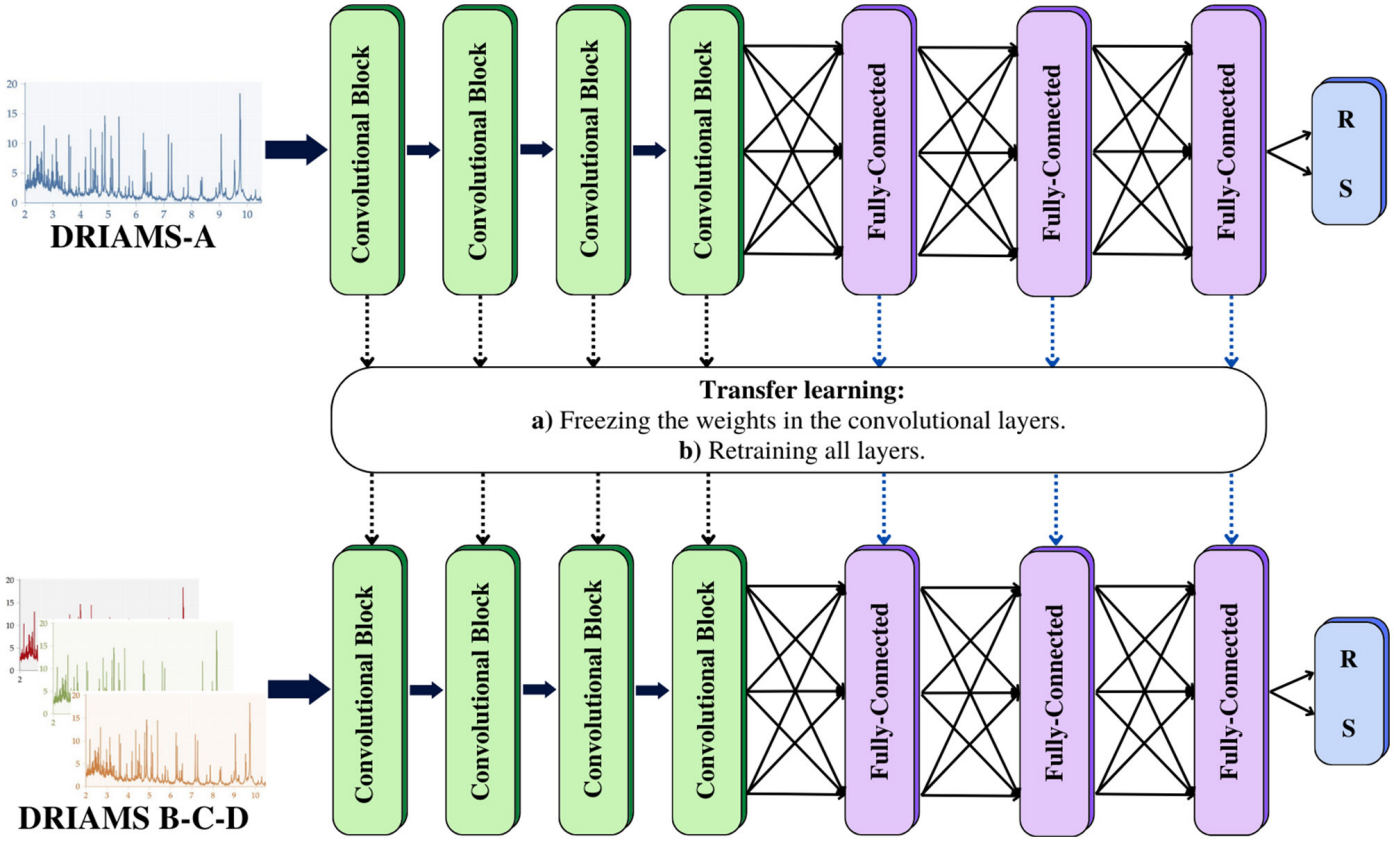
Transfer learning: a model is trained with a database containing an extensive number of samples. This model can be used as a starting point to be adapted to problems with similar characteristics. Transfer learning was implemented to evaluate our MSDeepAMR models on external databases. In the first case, we freeze the weights of the convolutional layers and only retrain the fully connected layers. For the second case, we update the weights of the entire model. R, Resistant; S, Susceptible.

In order to evaluate the potential benefits of transfer learning on MALDI-TOF data, this paper describes four experimental scenarios:

- Models trained and tested only on the external datasets.
- Evaluation of the best-performing model trained on DRIAMS-A when applied to the external data:

- Without transfer learning.
- Applying transfer learning, freezing the weights of the four convolutional layers, only retraining the weights of the fully connected layers (as shown in Figure 3).
- Applying transfer learning, retraining the weights of all layers.

In all cases, the same 20% of the external datasets were used to evaluate the model performances. In contrast, the remaining 80% were used to train the models (first scenario) or fine-tune the pre-trained model (transfer learning). An Adam optimizer was used to avoid overfitting with a learning rate of 10^−7^ for 10 epochs and a batch size of 32, sufficient for the model to fit the external data. The implementation of MSDeepAMR and examples of experiments found in this article are publicly available at: https://github.com/xlopez-ml/DL-AMR.

Furthermore, the evolution of the AUROC and AUPRC metrics has been studied when applying transfer learning by retraining all layers using different percentages (25%, 50%, 75%, and 100%) of the training set of the target datasets (DRIAMS–B–C–D) to study how models are affected by an increase of the number of samples available for the transfer learning.

#### 2.4.1 Feature importance analysis

In order to interpret the results obtained by the best-performing models, the analysis of SHAP values (using DeepExplainer) has been implemented to identify which *m/z* peaks are the most important when determining antimicrobial resistance or susceptibility. Specifically, how the most predominant peaks in the external datasets are affected before and after applying transfer learning will be analyzed.

### 2.5 Evaluation metrics

The main models trained with DRIAMS-A were implemented with a 10-fold cross-validation to avoid overfitting. Then, the transfer learning scenarios described in Section 2.3 were evaluated using ten random train-test splits. Therefore, the results reported in both cases are the mean of 10 iterations.

The metric Area Under the Receiver Operating Characteristic Curve (AUROC) and the Area Under the Precision-Recall Curve (AUPRC) were calculated. AUROC and AUPRC are metrics commonly used in binary classification problems of biological nature class (Chicco, 2017). The calculation of AUROC involves computing the area under the ROC curve, which represents the true positive rate or “recall” [Recall formula = (TP/TP+FN)] versus the false positive rate (1-specificity) [Specificity formula = (TN/TN+FP)]. This metric measures the model's discriminative ability, where a value of AUROC equal to 1 indicates a perfect model. In contrast, a value of 0.5 indicates performance similar to random guessing.

Regarding the AUPRC, this metric is calculated similarly but based on precision [Precision formula = (TP/TP+FP)] and recall, focusing on correctly classified positive values (minority class). AUPRC is a more reliable indicator for imbalanced datasets. We have also included the calculation of balanced precision, which consists of the arithmetic mean of sensitivity and specificity and is helpful in these cases. In the case of transfer learning, the metrics chosen to evaluate the models' performances also corresponded to AUROC and AUPRC.

## 3 Results

The main objective of this study was to implement models based on DL to develop our MSDeepAMR models that allow for the correct classification and identification of antibiotic resistance for different bacteria. All models were implemented from the raw MS data to improve the current state of the art, which was achieved with traditional machine learning algorithms (Wang et al., 2022; Weis et al., 2022; Zhang et al., 2022). As input data, we used raw mass spectra from the public database DRIAMS (Weis et al., 2022), selecting bacteria with the highest number of samples with antibiotic resistance profiles. Models were trained using DRIAMS-A and subjected to 10-fold cross-validation. Subsequently, each model was tested to evaluate its prediction performance using AUROC and AUPRC.

### 3.1 Results of MSDeepAMR models

**Figure 4:**
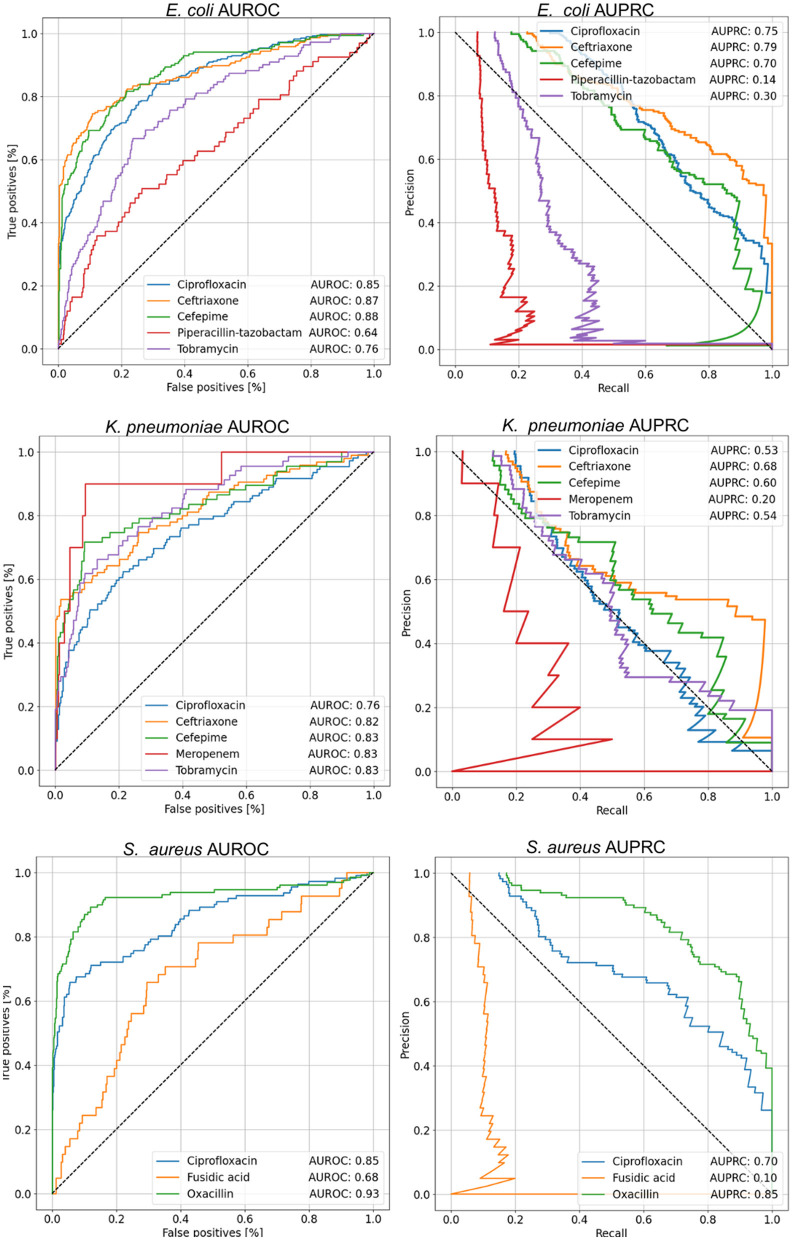
AUROC and AUPRC curves for each of the cases studied. The value shown in the tables within the figure corresponds to the mean of the 10-fold. About AUROC for *E. coli*, the best results were obtained for the antibiotics Ceftriaxone and Cefepime, while in *K. pneumoniae*, the best performance was achieved with Cefepime, Meropenem, and Tobramycin, with an AUROC of 0.83. On the other hand, in *S. aureus*, the best result was observed for Oxacillin. In terms of AUPRC, the highest performance in *E. coli* was achieved with Ceftriaxone. For *K. pneumoniae*, the best case was with Ceftriaxone, and finally, in *S. aureus*, the highest AUPRC value was obtained with Oxacillin.

To evaluate the classification performance of MSDeepAMR models, the AUROC, AUPRC, and balanced accuracy metrics were used. Models were implemented for different bacteria-antibiotic profiles: *E. coli* (ciprofloxacin, ceftriaxone, cefepime, piperacillin-T., tobramycin), *K. pneumoniae* (ciprofloxacin, ceftriaxone, cefepime, meropenem, tobramycin), and *S. aureus* (ciprofloxacin, fusidic acid, oxacillin). As shown in Figure 4, most models showed good performance (AUROC > 0.80), whereas the models for *E. coli, E. coli*-Ciprofloxacin, *E. coli*-Ceftriaxone, and *E. coli*-Cefepime showed an AUROC of 0.85, 0.87, and 0.88, respectively. On the other hand, analyzing the antimicrobial resistance profiles in *K. pneumoniae*, three of the five models implemented stand out: *K. pneumoniae*-Ceftriaxone, *K. pneumoniae*-Cefepime, and *K. pneumoniae*-Meropenem, which reach an AUROC of 0.82, 0.83, and 0.83, respectively. Finally, for *S. aureus* the *S. aureus*-Oxacillin model stands out with a good 0.93 AUROC. Regarding the AUROC, it is important to mention that for the study of resistance to Ciprofloxacin, the three bacteria presented a good performance, as shown in Figure 4, with an AUROC of 0.85 (*E. coli*-Ciprofloxacin), 0.76 (*K. pneumoniae*-Ciprofloxacin), and 0.85 (*S. aureus*-Ciprofloxacin), respectively.

Regarding the analysis of the results in the function of the AUPRC, it can be seen how the model is affected by the imbalance of classes in some cases. Therefore, the positive class must be more correctly classified, corresponding to the sample resistant to a given antibiotic. Nevertheless, our results showed that in *E.coli*, the better AUPRC corresponds to 0.75 (*E. coli*-Ciprofloxacin), 0.79 (*E. coli*-Ceftriaxone), and 0.70 (*E. coli*-Cefepime). In the case of *K.pneumoniae*, the best AUPRC was 0.68 for *K. pneumoniae*-Ceftriaxone. Finally, for *S. aureus* the better values of AUPRC were 0.70 (*S. aureus*-Ciprofloxacin) and 0.85 (*S. aureus*-Oxacillin).

Summarizing, regarding the global metrics of AUROC, AUPRC, and balanced accuracy shown in Table 2, the best model performances for each bacteria under study corresponded to *E. coli*-Ceftriaxone (AUROC 0.87, AUPRC 0.79, and B. acc 0.80), *K. pneumoniae*-Ceftriaxone (AUROC 0.82, AUPRC 0.68, and B. acc 0.76), and *S. aureus*-Oxacillin (AUROC 0.93, AUPRC 0.85, and B. acc 0.87).

**Table 2:** Performance results of 10-fold cross-validation in the ablation study and final MSDeepAMR model.

**Bacteria**	**Antibiotic**	**Baseline model**			**MSDeepAMR w/BN**			**MSDeepAMR w/BN + DO**		
		**B. Acc**	**AUROC**	**AUPRC**	**B. Acc**	**AUROC**	**AUPRC**	**B. Acc**	**AUROC**	**AUPRC**
*E. coli*	Ciprofloxacin	0.73 ± 0.03	0.84 ± 0.04	0.72 ± 0.03	0.73 ± 0.02	0.84 ± 0.04	0.73 ± 0.03	**0.74** **±0.01**	**0.85** **±0.03**	**0.75** **±0.03**
		Ceftriaxone	0.77 ± 0.02	0.87 ± 0.04	0.78 ± 0.03	0.78 ± 0.02	0.88 ± 0.05	0.78 ± 0.03	**0.80** **±0.01**	**0.87** **±0.03**	**0.79** **±0.03**
		Cefepime	0.75 ± 0.03	0.85 ± 0.02	0.65 ± 0.02	0.77 ± 0.03	0.87 ± 0.02	0.70 ± 0.03	**0.78** **±0.02**	**0.88** **±0.02**	**0.70** **±0.03**
		Piperacillin-T.	0.50 ± 0.03	0.58 ± 0.03	0.08 ± 0.04	0.50 ± 0.03	0.66 ± 0.03	0.12 ± 0.03	**0.51** **±0.04**	**0.64** **±0.04**	**0.14** **±0.05**
		Tobramycin	0.50 ± 0.03	0.69 ± 0.03	0.08 ± 0.04	0.52 ± 0.03	0.70 ± 0.02	0.26 ± 0.03	**0.55** **±0.03**	**0.76** **±0.02**	**0.30** **±0.04**
*K. pneumoniae*	Ciprofloxacin	0.50 ± 0.03	0.50 ± 0.03	0.18 ± 0.01	0.58 ± 0.02	0.70 ± 0.02	0.45 ± 0.03	**0.59** **±0.03**	**0.76+0.02**	**0.53** **±0.03**
		Ceftriaxone	0.73 ± 0.02	0.81 ± 0.02	0.67 ± 0.03	0.75 ± 0.01	0.82 ± 0.03	0.68 ± 0.04	**0.76** **±0.02**	**0.82** **±0.01**	**0.68** **±0.02**
		Cefepime	0.72 ± 0.04	0.76 ± 0.03	0.60 ± 0.04	0.69 ± 0.03	0.76 ± 0.02	0.57 ± 0.01	**0.75** **±0.01**	**0.83** **±0.01**	**0.60** **±0.03**
		Meropenem	0.50 ± 0.04	0.74 ± 0.03	0.12 ± 0.05	0.55 ± 0.02	0.73 ± 0.05	0.17 ± 0.04	**0.55** **±0.04**	**0.83** **±0.03**	**0.20** **±0.05**
		Tobramycin	0.63 ± 0.03	0.79 ± 0.02	0.46 ± 0.03	0.62 ± 0.02	0.78 ± 0.02	0.48 ± 0.01	**0.64** **±0.02**	**0.83** **±0.03**	**0.54** **±0.02**
*S. aureus*	Ciprofloxacin	0.67 ± 0.03	0.81 ± 0.02	0.57 ± 0.03	0.65 ± 0.02	0.82 ± 0.01	0.58 ± 0.04	**0.75** **±0.01**	**0.85** **±0.02**	**0.70** **±0.02**
		Fusidic acid	0.50 ± 0.03	0.50 ± 0.04	0.06 ± 0.04	**0.50** **±0.02**	0.67 ± 0.03	**0.13** **±0.04**	0.48 ± 0.04	**0.68** **±0.03**	0.10 ± 0.06
		Oxacillin	0.79 ± 0.03	0.90 ± 0.01	0.79 ± 0.04	0.79 ± 0.03	0.91 ± 0.02	0.81 ± 0.02	**0.87** **±0.01**	**0.93** **±0.02**	**0.85** **±0.01**

The best results for each pair of Bacteria-Antiobiotics are highlighted in bold font. The best global metric for each bacteria under study is highlighted in bold red font. B. Acc, balanced accuracy; BN, batch normalization; DO, dropout.

### 3.2 Ablation study

In order to obtain the most optimal and robust model, we evaluate the effect of batch normalization and dropout layers on the baseline model obtained after the hyperparameter search grid. A 10-fold cross-validation was applied for each of the 13 cases under study (Table 1). The experiments considered the following three different scenarios: (i) Baseline model; (ii) MSDeepAMR model with batch normalization; and (iii) MSDeepAMR model with batch normalization and dropout (final model).

As shown in Table 2, adding normalization and regularization layers improves the model's performance in most cases under study. Specifically, the best-performing models for each bacteria-antibiotic correspond to *E. col*i-Ciprofloxacin, *K. pneumoniae*-Ceftriaxone, and *S. aureus*-Oxacillin. Furthermore, using these layers in scenarios (ii) and (iii) improved the metrics by 1% to 2% and reduced the standard deviation. In other cases, when AUPRC values are low, regularization layers can substantially improve the model's performance. In detail, an example of this case corresponds to *K. pneumoniae*-Ciprofloxacin, where the AUPRC increases from 0.18 to 0.53 when the batch normalization and dropout layers are applied (Table 2).

### 3.3 External test and transfer learning results

The best-performing model was selected for each of the bacteria studied in the previous section, namely *E. coli*-Ceftriaxone, *K. pneumoniae*-Ceftriaxone, and *S. aureus*-Oxacillin. These models were tested with the external data subcollections (DRIAMS B-C-D). Subsequently, it was studied if implementing transfer learning improved the adaptation of the models to the external data. Table 3 shows the number of samples available in each case, where 80% was used for training and 20% for testing.

**Table 3:** Number of samples of each bacterium and antibiotic in external datasets (DRIAMS-D did not contain samples for the case *S. aureus*-Oxacillin).

**Dataset**	**Bacteria**	**Antibiotic**	**Susceptible**	**Resistant**
DRIAMS-B	*E. coli*	Ceftriaxone	168	45
		*K. pneumoniae*	Ceftriaxone	134	18
		*S. aureus*	Oxacillin	325	21
DRIAMS-C	*E. coli*	Ceftriaxone	765	151
		*K. pneumoniae*	Ceftriaxone	311	55
		*S. aureus*	Oxacillin	697	41
DRIAMS-D	*E. coli*	Ceftriaxone	1,796	198
		*K. pneumoniae*	Ceftriaxone	2,028	123
		*S. aureus*	Oxacillin	-	-

Tables 4–6 show the AUROC and AUPRC obtained in each of the transfer learning experiments described above. Regarding the *E. coli*-Ceftriaxone model (Table 4), the implementation of transfer learning achieves the best results of AUROC and AUPRC, where it is noted that DRIAMS-B had the best adaptability to the pre-trained model reaching an AUROC of 0.943, and an AUPRC of 0.752 in comparison to the 0.740 and 0.542 of AUROC and AUPRC obtained by training the model from scratch. For the *K. pneumoniae*-Ceftriaxone model (Table 5), the best results were also obtained with transfer learning, except in DRIAMS-C, where the model trained from scratch exceeded the AUROC and AUPRC obtained in the transfer learning experiment (0.594 vs. 0.512 in AUROC and 0.325 vs. 0.165 in AUPRC respectively). Finally, for the *S. aureus*-Oxacillin model (Table 6), in both DRIAMS-B and DRIAMS-C datasets, the transfer learning showed the best AUROC among the three experiments performed. Besides, it should be noted that in terms of AUPRC, the model's training from scratch presented better results than the tl test retraining all layers in the DRIAMS-B dataset (0.385 vs. 0.274, respectively). Besides, when studying the results obtained by retraining the neural network by freezing the weights of the convolution layers, in all cases, the results were lower than if we retrained the entire neural network.

**Table 4:** AUROC and AUPRC external testing and transfer learning of *E. coli*-Ceftriaxone model trained on DRIAMS-A.

**Experiment**	**DRIAMS-B**		**DRIAMS-C**		**DRIAMS-D**	
	**AUROC**	**AUPRC**	**AUROC**	**AURPC**	**AUROC**	**AUPRC**
Model trained with local data only	0.740	0.502	0.734	0.420	**0.764**	0.443
Test external data without tl	0.794	0.542	0.521	0.230	0.751	0.424
Test with tl freezing convolution layers	0.772	0.526	0.514	0.223	0.738	0.408
Test with tl retraining all layers	**0.943**	**0.752**	**0.741**	**0.463**	0.760	**0.571**

The best result for each case of study is highlighted in bold font.

**Table 5:** AUROC and AUPRC external testing and transfer learning of *K. pneumoniae*-Ceftriaxone model trained on DRIAMS-A.

**Experiment**	**DRIAMS-B**		**DRIAMS-C**		**DRIAMS-D**	
	**AUROC**	**AUPRC**	**AUROC**	**AURPC**	**AUROC**	**AUPRC**
Model trained with local data only	0.442	0.323	**0.594**	**0.325**	0.541	0.153
Test external data without tl	0.362	0.101	0.491	0.144	0.610	**0.204**
Test with tl freezing convolution layers	0.354	0.152	0.483	0.142	0.594	0.197
Test with tl retraining all layers	**0.571**	**0.353**	0.512	0.165	**0.653**	0.164

The best result for each case of study is highlighted in bold font.

**Table 6:** AUROC and AUPRC external testing and transfer learning of *S. aureus*-Oxacillin model trained on DRIAMS-A.

**Experiment**	**DRIAMS-B**		**DRIAMS-C**	
	**AUROC**	**AUPRC**	**AUROC**	**AURPC**
Model trained with local data only	0.683	**0.385**	0.654	0.075
Test external data without tl	0.724	0.181	0.674	0.143
Test with tl freezing convolution layers	0.717	0.175	0.642	0.281
Test with tl retraining all layers	**0.793**	0.274	**0.782**	**0.302**

The best result for each case of study is highlighted in bold font.

The results of the analysis increasing the amount of target data used for the fine-tuning are shown in Supplementary Figure S1. DRIAMS-B was the subset that best adapted to the models trained on DRIAMS-A, despite being the one with the smallest number of samples available for training. On the other hand, the DRIAMS-C and D subsets show that, despite not having obtained significant improvements in the prediction accuracy, it improves consistently along with the number of samples used in the model fine-tuning. In this way, the results of this experiment show that as the percentage of samples increases, the AUROC and AUPRC also improve, demonstrating that a small amount of new samples can have a large impact on the model's performance after fine-tuning.

Respecting the feature importance analysis, the SHAP values results are shown in Supplementary Figures S2–S5. SHAP values were computed for the three best models obtained for each bacteria under study: *E. coli*-ceftriaxone (Supplementary Figure S3), *K. pneumoniae*-ceftriaxone (Supplementary Figure S4), and *S. aureus*-oxacillin (Supplementary Figure S5). The SHAP values were computed on DRIAMS B, C, and D in order to analyze the impact of the most important features (*m/z* peaks) in the fine-tuning process.

Analyzing the results obtained on DRIAMS-A (Supplementary Figure S2), it can be seen that the proposed model focuses the attention on the first part of the spectrum (2,000Da–7,000Da), which contains ions of lower mass, which separate easily, allowing for better differentiation between spectra of susceptible and resistant bacteria.

In the case of *E. coli*-Ceftriaxone, when the model is tested on DRIAMS-B (Supplementary Figure S3A), it is observed that most of the *m/z* peaks appear in the range 6,800–6,900Da, but after the transfer learning, they become closer to those of the base model. It is important to note that when transfer learning is applied, the 8,450 Da peak appears among the top 20 features, previously attributed to antibiotic multi-resistance in *Escherichia coli*. For the DRIAMS-C (Supplementary Figure S3B) and D (Supplementary Figure S3C) cases, there are no major differences with respect to the base model, except that for the DRIAMS-C case where some peaks in the range (6,800–6,900 Da) also stand out, but their direct relationship with antibiotic resistance has not been documented yet.

For the case of *K. pneumoniae*-Ceftriaxone, the tendency of the base model remains similar: a large part of the most important peaks are present in the range of 2,000–3,000 Da. However, when testing external datasets (Supplementary Figure S4), it is observed that these spectra focus their differentiation on the *m/z* peaks 7,770–4,736–2,135–7,706 Da, which, together with other peaks, coincide with those reported by Weis et al. (2022) which could help to confirm their relationship with the identification of antimicrobial resistance.

Finally, for the case of *S. aureus*-Oxacillin, in the base case (DRIAMS-A, Supplementary Figure S5A), the absence of the *m/z* peaks 2,414 Da (PSM-mec) and 3,006 Da (agr-positive), which have been widely documented to be directly attributable to the MRSA subspecies (methicillin-resistant Staphylococcus aureus), stands out. When analyzing the SHAP values for the DRIAMS-B dataset (Supplementary Figure S5B), the identification of peak 2,414 stands out in this case, along with the appearance of peak 4,517, also reported by Weis et al. (2022) and previously associated with antibiotic resistance [MRSA clonal complexes (CC398)]. In the case of DRIAMS-C (Supplementary Figure S5C), some of the peaks previously associated with antibiotic resistance do not stand out, but *m/z* peaks 2,411 and 2,417 Da are found, which could be associated with peak 2,414 Da in relation to calibration differences in the equipment used.

## 4 Discussion

In this study, MSDeepAMR models based on DL were implemented in order to predict AMR. Specifically, the MSDeepAMR model was applied on three different bacteria with varied antibiotic resistance profiles: *E. coli* (ciprofloxacin, ceftriaxone, cefepime, piperacillin-T., tobramycin), *K. pneumoniae* (cefepime, ciprofloxacin, ceftriaxone, meropenem, tobramycin), and *S. aureus* (oxacillin, ciprofloxacin, fusidic acid). Raw MS data were used, and deep learning methods were applied to obtain MSDeepAMR models. Out of the trained models, the best AUROC and AUPRC metrics performances were obtained for the following models: *E. coli*-Ceftriaxone, *K. pneumoniae*-Ceftriaxone, and *S. aureus*-oxacillin (Table 2). Subsequently, these models were used to study their adaptability to external data (Table 3). As for the remaining models, we consider that lower performances of AUPRC are due to the predominant class imbalance in the datasets, so future research should focus on developing methodologies to build robust classifiers to the predominant class imbalance in the study of antibiotic resistance.

Table 7 show the results obtained with MSDeepAMR, comparing our results with state-of-the-art machine learning algorithms, and specifically with the research of Weis et al. (2022). A Wilcoxon test was applied and detected statistically significant differences (*p*-value < 0.05) between our MSDeepAMR model and the state-of-the-art results. In detail, considering that the data used were the same, it can be seen in Table 7 that the MSDeepAMR model improves the AUROC values obtained by an average of 13% compared to the more traditional machine learning algorithms implemented by Weis et al. (LightGBM for *E. coli*, Multi-layer perceptron for *K. pneumoniae* and LightGBM for *S. aureus*). As for the AUPRC, the performance of our model considerably exceeded the results obtained in the previous research, even doubling the AUPRC obtained in the best cases, as was the case for *E. coli*-Ceftriaxone (0.79 vs. 0.30), *E. coli*-Cefepime (0.70 vs. 0.24), and *K. pneumoniae*-Ceftriaxone (0.68 vs. 0.33).

**Table 7:** MSDeepAMR performance results, comparing the present study and the previously obtained by the state of art (Weis et al.).

**Bacteria**	**Antibiotic**	**AUROC**		**AUPRC**	
		**MSDeepAMR**	**Weis et al**.	**MSDeepAMR**	**Weis et al**.
*E. coli*	Ciprofloxacin	**0.85**	0.76	**0.75**	0.60
		Ceftriaxone	**0.87**	0.74	**0.79**	0.30
		Cefepime	**0.88**	0.73	**0.70**	0.24
		Piperacillin-T.	**0.64**	0.60	**0.14**	0.10
		Tobramycin	**0.76**	0.64	**0.30**	0.18
*K. pneumoniae*	Ciprofloxacin	**0.76**	0.68	**0.53**	0.31
		Ceftriaxone	**0.82**	0.74	**0.68**	0.33
		Cefepime	**0.83**	0.76	**0.60**	0.31
		Meropenem	**0.83**	0.55	**0.20**	0.16
		Tobramycin	**0.83**	0.74	**0.54**	0.29
*S.aureus*	Ciprofloxacin	**0.85**	0.72	**0.70**	0.43
		Fusidic acid	**0.68**	0.65	0.10	**0.13**
		Oxacillin	**0.93**	0.80	**0.85**	0.49

The best result for each case of study is highlighted in bold font.

Concerning the ablation study, it is worth mentioning that normalization and regularization layers constitute a fundamental part of the neural network architecture for this type of data, as shown in Table 2; the use of these layers improved the results obtained in most of the cases presented.

Regarding the implementation of transfer learning or domain adaptation methodologies, we found that, although the equipment used for sample collection in each laboratory belonged to the Microflex Biotyper System by Bruker Daltonics product family, adapting a pre-trained model to data from a new laboratory is not a simple task. It is partially due to the high number of genetic and biological factors that distinguish bacterial strains according to their origin or slight differences in sample collection parameters. Nevertheless, it was demonstrated by the experiments performed that retraining all layers of a model to adjust it to data from a new laboratory is a better starting point than training a model from scratch. These promising results open the way for further research on transfer learning in models that include MALDI-TOF mass spectrometry data.

Besides, it was demonstrated that when the sample size increases, the transfer learning results improve considerably (Supplementary Figure S1). This implies that our methodology enables AMR detection even when there is a very small amount of data, although the availability of a larger number of samples can improve the model's performance.

Finally, it was demonstrated that when a large number of samples (over 3,000) are available, it is possible to generate deep-learning models with high performance in identifying resistance or susceptibility to a given antibiotic. These models can be used in clinical routines to quickly and efficiently identify the optimal treatment to be implemented, avoiding the wait for traditional bacterial cultures and the indiscriminate use of broad-spectrum antibiotics.

## 5 Conclusion

This work proposes a complete methodology for antimicrobial resistance prediction from raw mass spectrometry data. An approach based on deep learning was applied. Deep learning is designed to identify patterns in complex and extensive data. In our case, MS data with their *m/z* peaks allow us to characterize whether a bacterium is resistant or susceptible to an antibiotic. To demonstrate the effectiveness of this approach, the mass spectra of *Escherichia coli, Klebsiella pneumoniae*, and *Staphylococcus aureus* bacteria were analyzed in concordance with their AST profiles. The datasets were constructed based on a recently published free database (Weis et al., 2022). Our results showed that the implemented MSDeepAMR models were efficient and effective for AMR prediction on this type of data. Furthermore, our MSDeepAMR models showed better performance (AUROC) than the state of art results (Wang et al., 2022; Weis et al., 2022; Zhang et al., 2022). Besides, those studies are made with traditional machine algorithms. Since deep learning models require a significant number of samples for training, a complication for laboratories with a low sample collection rate, the implementation of transfer learning was studied.

Transfer learning results demonstrated that the developed MSDeepAMR models could be used for other laboratories as a starting point to adapt them to their data, guaranteeing the reproducibility of our models. Besides, our results showed that MSDeepAMR models allow the correct work of raw MS data. The MSDeepAMR models gave good results in classification and prediction. In addition, transfer learning will allow using these models on new samples to provide reproducibility, which is necessary for this area when predicting AMR in different laboratories. Nevertheless, it is still required to continue optimizing the methodologies for antimicrobial resistance analysis from MALDI-TOF mass spectra and to continue contributing to the creation of public databases from different laboratories worldwide.

Finally, one limitation is that we consider MALDI-TOF from Bruker, which produces data with a different length dimension in comparison to other equipment, for example, with the MALDI-TOF from bioMrieux. In future research, adaptations of this methodology to inputs from other MALDI-TOF devices may be explored, potentially opening the door to cross-device AMR models.

In future work, MSDeepAMR within the MALDI-TOF device could be used to enable the on-the-fly AMR detection because the proposed network allows the classification of the raw data directly, which is an advantage because it avoids any manual preprocessing. In this study, three main bacteria in the DRIAMS dataset were studied. Nevertheless, the methodology could be evaluated on more bacteria/antibiotic pairs. For this purpose, we published our code as open-source to enable other researchers and practitioners to extend this line of research.

## Data availability statement

The original contributions presented in the study are included in the article/Supplementary material, further inquiries can be directed to the corresponding author.

## Author contributions

XL-C: Writing – review & editing, Writing – original draft, Validation, Supervision, Resources, Project administration, Methodology, Investigation, Funding acquisition, Conceptualization. JM-T: Writing – review & editing, Writing – original draft, Visualization, Validation, Software, Methodology, Formal analysis, Data curation. RH-G: Writing – review & editing, Writing – original draft, Supervision, Resources, Methodology, Conceptualization. DP: Writing – review & editing, Writing – original draft, Validation, Supervision, Methodology, Investigation, Conceptualization.
