# Supplementary material for: MSDeepAMR: antimicrobial resistance prediction based on deep neural networks and transfer learning
Source: Front Microbiol. 2024 Apr 17;15:1361795. doi: 10.3389/fmicb.2024.1361795 (PMC11062410; doi:10.3389/fmicb.2024.1361795)

## Supplementary Material

**Fig. S1.** AUROC and AUPRC at different percentages of samples (25%, 50%, %75%, and 100%) tested among the target datasets DRIAMS B, C, and D.

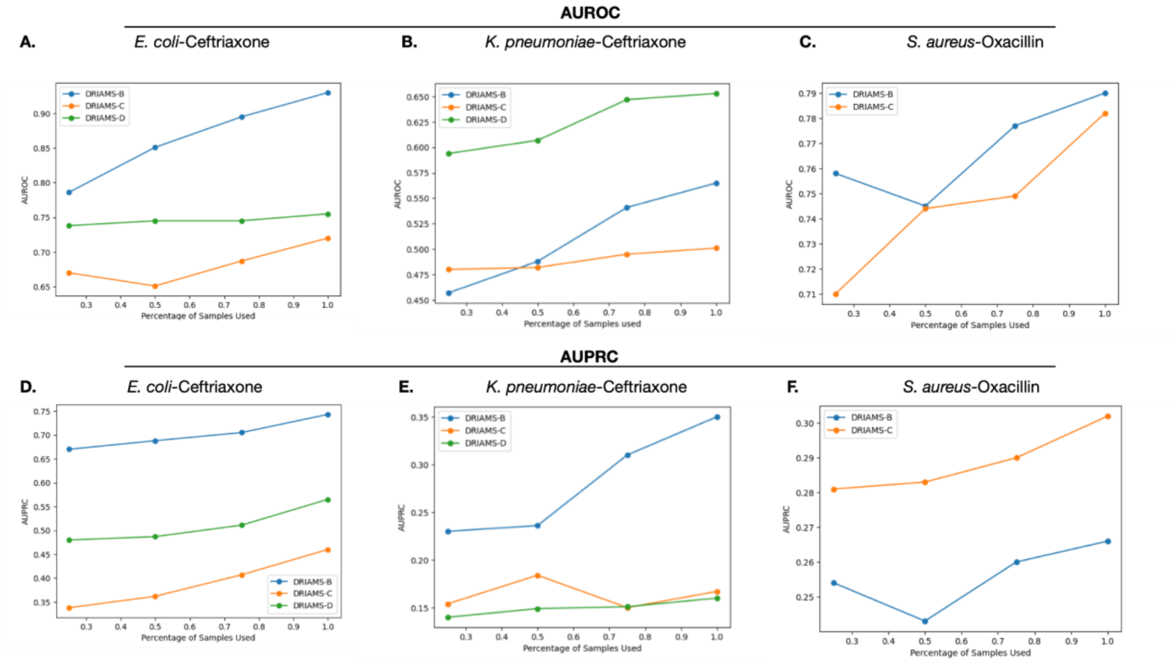

**Fig. S2.** Mean absolute SHAP values results for the best 3 models *E. coli*-Ceftriaxone, *K. pneumoniae*-Ceftriaxone, and *S. aureus*-Oxacillin, in DRIAMS A.

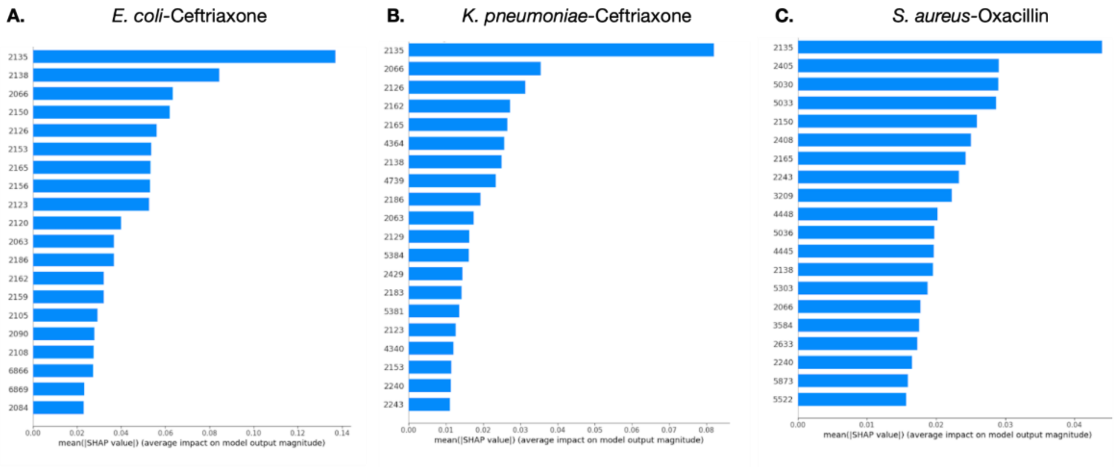

**Fig. S3.** Mean absolute SHAP values results for *E. coli*-Ceftriaxone among DRIAMS B (S3.A), C (S3.B), and D (S3.C), without and with TL.

A

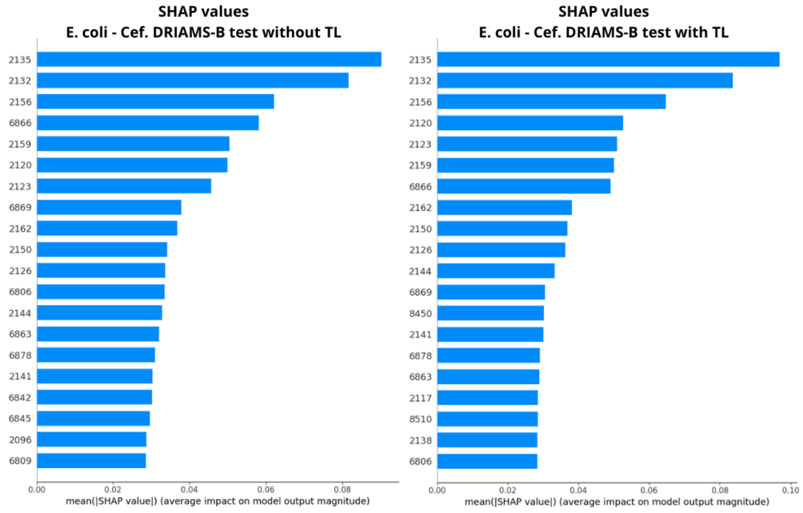


B

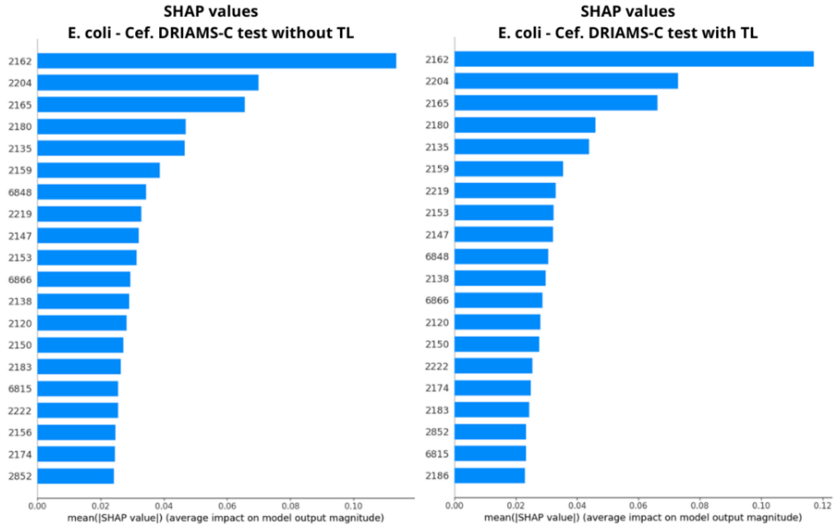


C

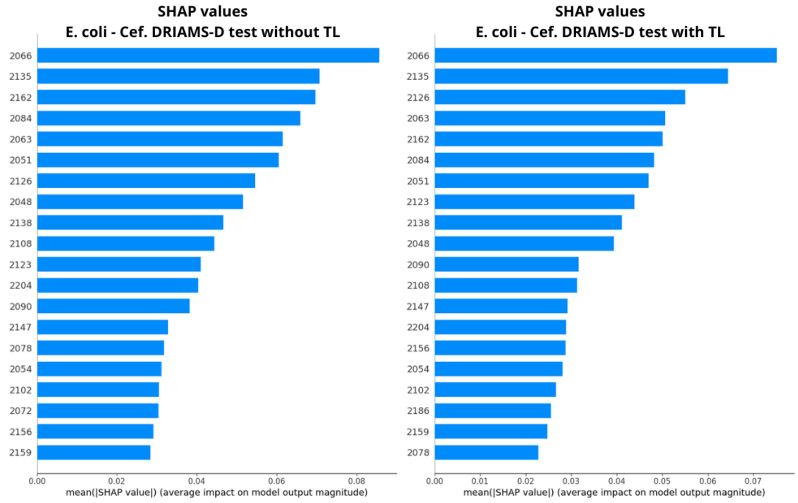

**Fig. S4.** Mean absolute SHAP values results for *K. pneumoniae*-Ceftriaxone among DRIAMS B (S4.A), C (S4.B) and D (S4.C), without and with TL.

A

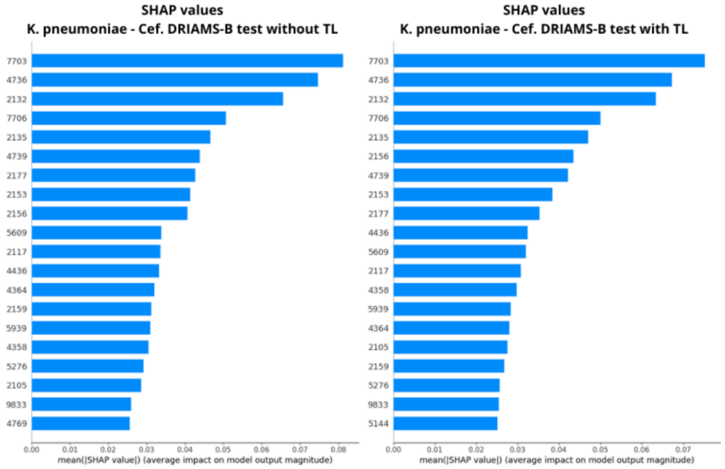


B

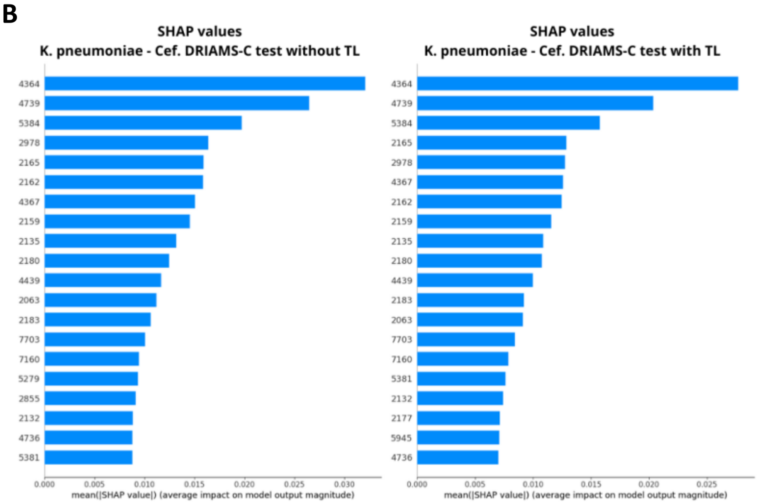


C

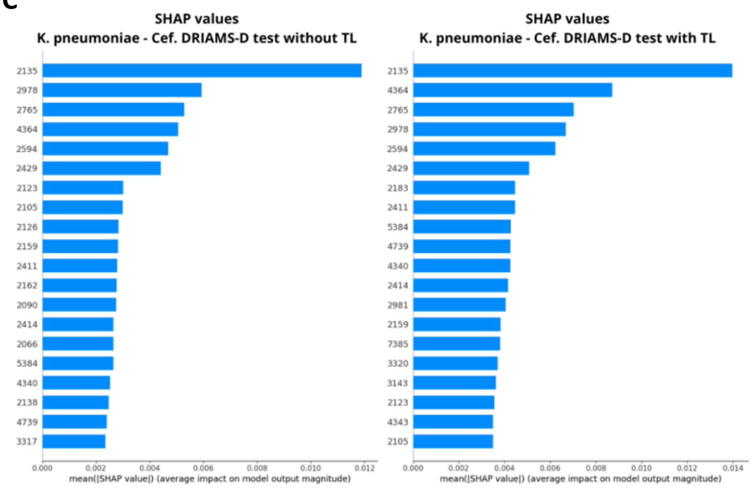

**Fig. S5.** Mean absolute SHAP values results for *S. aureus*-Oxacillin among DRIAMS B (S5.A) and C (S5.B) without and with TL.

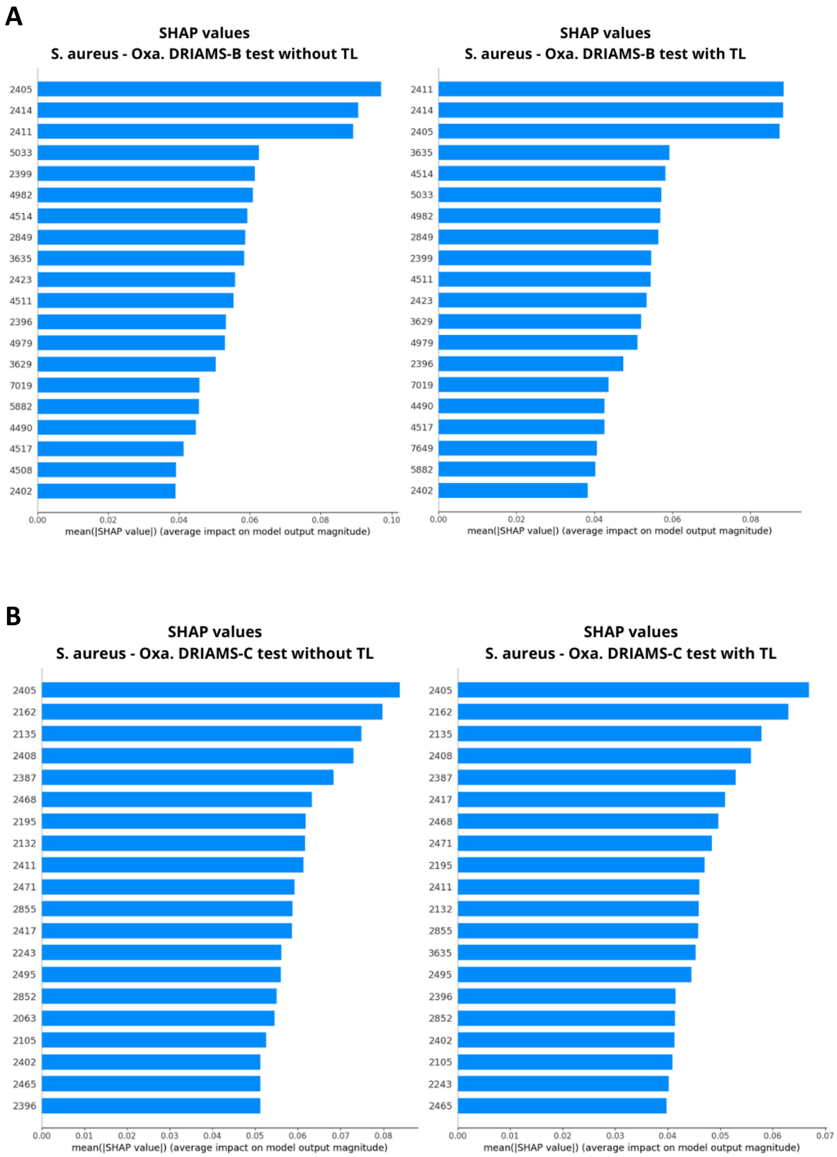

Supplement: Supplementary file 1 [file Data_Sheet_1.PDF]
